# Immune Checkpoint Inhibitors: A New Opportunity in the Treatment of Ovarian Cancer?

**DOI:** 10.3390/ijms17071169

**Published:** 2016-07-20

**Authors:** Gloria Mittica, Sofia Genta, Massimo Aglietta, Giorgio Valabrega

**Affiliations:** 1 Candiolo Cancer Institute-FPO-IRCCS, Candiolo, Turin 10060, Italy; gloria.mittica@ircc.it (G.M.); sofia.genta@ircc.it (S.G.); massimo.aglietta@ircc.it (M.A.); 2 Department of Oncology, University of Torino, Turin 10060, Italy

**Keywords:** ovarian cancer, immune checkpoint inhibitor, ipilimumab, nivolumab, pembrolizumab, cytotoxic T-lymphocyte-associated protein 4 (CTLA-4), programmed cell death-1 (PD-1), programmed cell death ligand-1 (PD-L1), tumor infiltrating lymphocytes (TILs)

## Abstract

Epithelial ovarian cancer (EOC) is the leading cause of death for gynecological cancer. The standard treatment for advanced stage is the combination of optimal debulking surgery and platinum-based chemotherapy. Nevertheless, recurrence is frequent (around 70%) and prognosis is globally poor. New therapeutic agents are needed to improve survival. Since EOC is strongly immunogenic, immune checkpoint inhibitors are under evaluation for their capacity to contrast the “turn off” signals expressed by the tumor to escape the immune system and usually responsible for self-tolerance maintenance. This article reviews the literature on anti-cytotoxic T-lymphocyte-associated protein 4 (CTLA-4), anti-PD-1, anti-PD-L1, and anti-PD-L2 antibodies in EOC and highlights their possible lines of development. Further studies are needed to better define the prognostic role of the immune checkpoint inhibitors, to identify predictors of response and the optimal clinical setting in EOC.

## 1. Introduction

Epithelial ovarian cancer (EOC) is the first cause of death among gynecological neoplasms and the fifth cancer-related cause of death for women in advanced countries [1]. Cytoreductive surgery integrated with platinum-based chemotherapy is the standard treatment for advanced EOC. Despite this combined approach, most patients recur and eventually die, with prognosis significantly depending on the platinum-free interval. Usually, platinum-sensitive disease benefits from platinum-based re-challenging, while platinum-resistant disease is usually treated with single-agent chemotherapy (e.g., pegylated liposomal doxorubicin [2,3], paclitaxel [4], gemcitabine [2,3], etoposide [5], topotecan [6,7], or docetaxel [8]).

Several drugs have been recently added to the already available armament, such as bevacizumab and olaparib, for BRCA-mutated EOCs. Unfortunately, their contribution to survival improvement of patients with relapsed EOC, is, at best, marginal and, overall, prognosis remains globally severe [9,10,11].

New effective therapeutic strategies are needed to overcome drug resistances, preventing spreading and progression of the tumor.

In this review, we will focus on the current clinical evidence and future perspectives offered by immune checkpoint inhibitors in EOC. To identify ongoing clinical trials with these molecules, we operated a search on clinicaltrials.gov with the keywords “ovarian cancer” and the name of each checkpoint inhibitor discussed in the article.

## 2. Immune Checkpoint Inhibitors: Prognostic Value and Rationale for Clinical Use

In recent years there is an increasing interest related to the role played by adaptive and innate immunity in cancer progression control. It has been demonstrated that immune cells, through the recognition of tumor-associated antigens (TAAs) and tumor-specific antigens (TSA), are able to identify and kill neoplastic cells [12]. Ovarian cancer is known to be an immunogenic tumor. In 2003 Zhang and colleagues [13] observed that the presence of CD3+ tumor-infiltrating lymphocytes (TILs) is correlated with improved survival in EOC patients. Two years later, Sato and colleagues [14] reported an improved survival in patients with intraepithelial CD8+ tumor infiltrating lymphocytes and a high CD8+/regulatory T cell ratio. While the presence of CD8+ infiltrating lymphocytes seems to be a good predictor of prognosis, the presence of CD4+CD25+FoxP3+ T regulatory cells (Tregs) and the existence of a natural killer (NK) and B cell infiltration seems to be correlated with a worse prognosis [15,16].

In the physiological immune response, after TAA recognition by CD4+ and CD8+ T cells, processed into small peptide and presented by antigen-presenting cells (APCs) through major histocompatibility complex (MHC) class II, two positive signals are required for activation. The first one is the connection between the T cell receptor (TCR) and MHC molecules; the second necessary step is the interaction between B7 on APCs and CD28 on T cells. The CD28 has a competitive receptor for B7 ligand, the cytotoxic T lymphocyte antigen-4 (CTLA-4), which delivers an inhibitory signal. While this negative feedback is mainly used in secondary lymphoid organ, other inhibitory pathways are present within the tumor microenvironment [17,18]. The most important peripheral regulatory pathway is the interaction between the programmed cell death-1 (PD-1) receptor, expressed on T cells, and the programmed cell death ligand-1 and 2 (PD-L1 and PD-L2) on the tumor cells surface.

**Figure 1:**
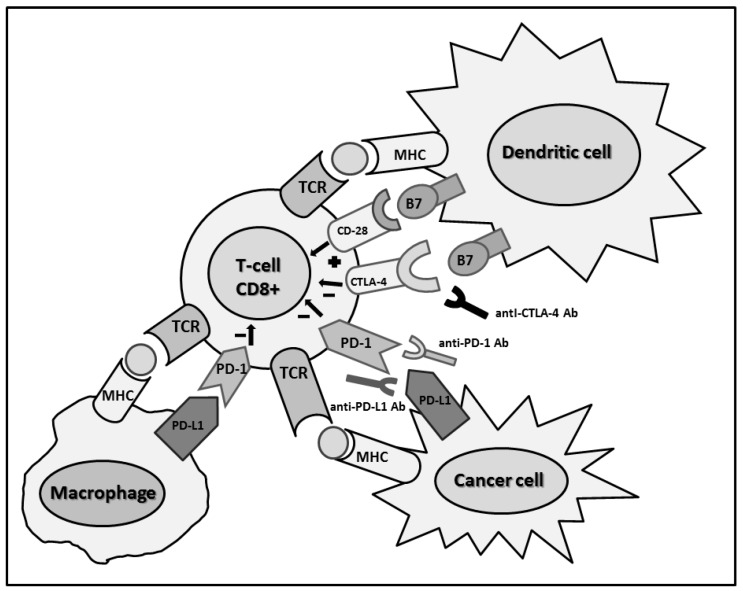
Cytotoxic T-lymphocyte-associated protein 4 (CTLA-4) and PD-1/L1 checkpoint blockade. MHC, major histocompatibility complex; TCR, T cell receptor; PD-1, programmed cell death-1; PD-L1, programmed cell death ligand-1; +, B7 receptor expressed; −, B7 receptor not expressed.

The binding between PD-1 and PD-L1 causes the inhibition of T-cells proliferation, the cytokine’s secretion, and the increase of Treg, ensuring the maintenance of self-tolerance [19,20]. A similar immunosuppressive effect is determined by the binding between PD-1 and the second ligand called PD-L2 (Figure 1) [21]. This control mechanism, usually used by epithelial cells and leukocytes, and induced by interferon-γ (IFN-γ) secretion, is necessary to prevent the development of autoimmune reactions.

Tumor cells are able to evade immune surveillance through different mechanisms and one of these is the exposure of PD-L1 [22,23]. A high expression of PD-L1 seems to be correlated with a worse prognosis in several type of tumors, such as non-small cell lung cancer (NSCLC) [24,25], kidney cancer [26,27,28,29,30,31], and bladder cancer [32], while in other neoplasms, like melanoma [33], seems to be a good prognosis predictor.

In ovarian cancer, Hamanishi and colleagues [34] observed a significant inverse correlation between the intraepithelial CD8+ T lymphocyte count and the expression of PD-L1 in tumor cells. Moreover, they reported a significantly worse prognosis for patients with higher levels of PD-L1 in tumor cells. More recently, other authors have investigated the role of PD-L1 expression as a prognostic factor in EOC. In 2013 Maine and colleagues [35] observed a correlation of PD-1 expression with malignant tumors versus benign/borderline, and they also found that PD-L1 expression on monocytes in ascites and blood of malignant EOC patients is higher than those with benign/borderline disease. Not all studies agree in attributing the role of negative prognostic factors to the expression of PD-1 and PD-L1. Darb-Esfahani and colleagues [36] reported a significantly increased progression free survival (PFS) in patients with higher PD-1 and PD-L1 expression in cancer cells, CD3+, PD-1+, and PD-L1+ TILs densities, as well as PD-1 and PD-L1 mRNA levels. PD-L1 expression has, also, significant impact on overall survival (OS) (*p* = 0.045), while for PD-1 expression only a positive trend was seen for OS (*p* = 0.059) [36]. These data has been confirmed in a recent article published by Webb and colleagues [37]. Probably, discordant results may reflect different techniques for PD-L1 and PD-1 assessment.

Independently from the prognostic significance of PD-L1 expression, PD-L1/PD-1 receptor B7/CTLA-4 interactions are important immune escape mechanisms, allowing tumor progression.

In order to prevent the activation of the immune-inhibitory pathways, several monoclonal antibodies are under development. Currently, various antibodies targeting PD1, PD-L1, PD-L2, and CTLA-4 have shown activity in several cancer other than EOC, such as melanoma [38,39,40,41], lung cancer [42,43,44], head cancer, and renal cell carcinoma [45]. In melanoma and non-small cell lung cancer, immune checkpoint inhibitors have been approved.

## 3. CTLA-4 and PD-1/PD-L1 Blockade in Ovarian Cancer: Clinical Evidence

Ipilimumab is a fully human immunoglobulin class G1 (IgG1) antibody targeting CTLA-4 and it is currently approved for the treatment of metastatic melanoma [46]. Between 2003 and 2008 Hodi et al. [46], in a two-steps study, administered ipilimumab to eleven stage IV ovarian cancer patients, previously vaccinated with granulocyte-macrophage colony-stimulating factor (GM-CSF) modified irradiated autologous tumor cells (e.g., GVAX). One out of nine patients of the second group obtained a durable disease control (over 4 years), while three patients had a disease stabilization. Tumor regression was correlated with CD8+/Treg ratio suggesting a potential synergistic role of the association of anti-CTLA-4 with the Treg depleting therapies. The safety profile was favorable and only two patients experienced grade 3 gastrointestinal toxicities [47,48].

The other anti CTLA-4 antibody in an advanced stage of development is tremelimumab. For this fully-human IgG2 antibody no clinical evidence is yet available for EOC, but several studies are ongoing (see the next section). The first anti-PD-1 tested in EOC was nivolumab, a fully-humanized IgG4, which prevents the binding between PD-1 and its ligands.

In a phase II trial published by Hamanishi and colleagues [49], nivolumab was administered in two cohorts of patients at a dose of 1 or 3 mg/kg. All of the women included in the study had platinum-resistant EOC and they had already received at least two chemotherapy lines. Two complete responses (CRs) were observed in the 3 mg/kg arm and one partial response occurred in the 1 mg/kg arm. Considering both cohorts, overall response rate (ORR), was 15%, and the disease control rate (DCR) was 45% [49]. One of the two CRs occurred in a patient with a clear cell carcinoma (CCC), usually resistant to chemotherapy [50]. Most tumor specimens (80%) showed high expression of PD-L1, but no significant correlated with response was observed. Eight out of 20 patients enrolled (40%) developed grade 3 or 4 treatment-related adverse events. The most common were hypothyroidism, lymphocytopenia, fever, transaminitis, rash, fatigue, anemia, arthralgia, and arrhythmia [49].

Varga and colleagues [51] presented the interim first results of a phase Ib trial, evaluating safety and antitumor activity of pembrolizumab (formerly known as lambrolizumab), another anti-PD-1 antibody, in patients with PD-L1-positive advanced solid tumors (PD-L1 expression ≥1%). One out of the 26 patients with advanced EOC obtained a complete response, while two patients experienced a partial response. The best overall response rate was 11.5% and most common adverse events reported were fatigue, anemia, and decreased appetite.

In 2012 Brahmer et al. [52] treated 207 patients with advanced solid tumors, including 17 women with ovarian cancer, with BMS-936559, a fully-human IgG4 antibody targeting PD-L1. One patient had a partial response and three had a stable disease. Most common toxicities were fatigue, infusion reaction, diarrhea, arthralgia, rash, rash, pruritus, and headache.

In a phase Ib trial, Disis and colleagues treated 124 women affected by recurrent or refractory EOC with avelumab, a fully-humanized anti-PD-L1 IgG1 antibody, at the dose of 10 mg/kg. ORR was 9.7% and 12 partial responses have been reported. Fifty five patients experienced a stable disease (44.4%) and the disease control rate (DCR) was 54%. Immune-related adverse events have been reported in 66.1%, with 6.5% grade ≥3 (increased lipase and elevated creatine kinase and autoimmune myositis that led to interruption of treatment). The most frequent adverse events were fatigue, nausea, and diarrhea [53].

## 4. Ongoing Studies

In all published trials, immune checkpoint inhibitors were tested as single agents in relapsed platinum resistant EOC with ORRs not exceeding 15% and only a few patients showing long-lasting disease control. In order to improve these results, the combination with other agents, first of all chemotherapy, is under investigation. Interestingly, Grabosch et al. [54], in a preclinical model, reported that chemotherapy exposure with platinum and taxane upregulates PD-L1. Moreover, combination of high-dose PD-L1 inhibitor and cisplatin in vivo is able to control and reduce tumor burden (*p* = 0.029), even if optimal timing and dosage are not yet defined [54,55,56].

Following evidence derived from metastatic melanoma, several trials are also investigating associations between different checkpoint inhibitors.

However, probably the most promising strategy, in the subgroup of BRCA-mutated/Homologous recombination deficient (HRD) + EOC is the association of immune checkpoint inhibitors with poly ADP-ribose polymerase (PARP)-inhibitors (see Table 1). Preclinical data indicate that BRCA-deficient ovarian cancers express more frequently immune response genes than wild-type tumors [57]. On these bases, in a murine ovarian cancer model Higuchi and colleagues [58] demonstrated that the CTLA-4 antibody, but not the inhibition of the PD-1/PD-L1 pathway, synergize with PARP-inhibitors increasing long term survival in the majority of mice (*p* < 0.0001). Possibly, long-term results are due to a T-cell mediated response and to the development of a protective immune memory.

## 5. Discussion and Future Perspectives

The treatment of advanced/relapsed EOC is clearly an unmet need. Immune checkpoint inhibitors may improve clinical outcome, but before adding them to our therapeutic options several questions need to be addressed:

(1) Do we have reliable predictors of response in EOC?
(2) Are there subgroups more likely to benefit from immune checkpoint inhibitors?
(3) Is there an optimal clinical setting?
(4) Is it better to use checkpoint inhibitors alone or in association with other agents?
(5) Is there a possible role for PD-L1/L2 in early detection of ovarian cancer?

(1) The most important point is the lack of validated predictive biomarkers of response. Unfortunately, the expression of PD-L1 on tumor cells does not seem to be a reliable predictor of benefit from immune checkpoint inhibitors since its role is not consistent across different studies in different cancers probably due heterogeneous techniques in the measurement of PD-L1 and different timing of assessments (e.g., before chemotherapy, after chemotherapy, or during chemotherapy) [59]. Moreover, it has to be taken into account that, other non-neoplastic cells in the tumor microenvironment expressing PD-L1 may be relevant in response to checkpoint inhibitors. For example, Webb and colleagues have shown, in ovarian cancer, that tumor-associated macrophages (TAMs) frequently express PD-L1 [37].

Considering predictors less “PD-1/PD-L1 and PD-L2centric” and more easily applicable to clinical practice, there are a few interesting reports on correlations between the anti CTLA-4 ipilimumab and response in metastatic melanoma that may future relevance also in EOC The most interesting are the increased number of peripheral blood absolute lymphocyte count [60] and the low ratio value of absolute neutrophils and lymphocyte count (N/L) [61].

(2) A peculiar subset of EOC patients is represented by somatic and germline BRCA mutation carriers. It is known that EOCs developed in BRCA carriers are characterized by a higher mutational load and there is some evidence that this determines changes in the tumor microenvironment due to the greater number of neoantigens that favor recruitment of an increased number of TILs, as for other hypermutated tumors [62,63]. In fact, BRCA-mutated EOCs seem to be associated with increased CD3+ and CD8+ TIL counts and high levels of PD-1 and PD-L1 [64,65]. These data suggest that BRCA-mutated patients may represent a particularly favorable subset for immunotherapy in general and, in particular, for immune checkpoint inhibitors, alone or, even more likely, in association with PARP inhibitors or platinum-based chemotherapy. Additionally, rare histologies, which are known to be poorly sensitive to chemotherapy, may represent a favorable subset for immune therapy [66].

In particular, clear cell ovarian cancer (CCC) is characterized by an intrinsic chemoresistance. At American Society of Clinical Oncology (ASCO) 2016 it was shown that CCCs are frequently associated with microsatellite instability (MSI) leading to a higher number of CD3+TILs and PD-1+TILs in comparison with high-grade serous ovarian cancer (HGSOC) [67]. Moreover, CCCs have a gene expression profile similar to renal cancer and it is known that a high rate of alterations in the PI3K/Akt/mTOR pathway characterizes this histological type [68]. Interestingly, preclinical data from NSCLC suggest that the activation of this pathway correlates with an increased expression of PD-L1 in tumor cells [69].

These considerations support the clinical observation of a complete response (CR) obtained with nivolumab, reported in a CCC by Hamanishi and colleagues [49,70].

(3) Another important point that needs that to be discussed is the optimal clinical setting for the use of immune checkpoint inhibitors: from the few clinical trials available, platinum-resistant EOCs, which are characterized by unfavorable prognosis and general chemoresistance, seem a reasonable target. However, lower tumor burden, which usually identifies platinum-sensitive disease, may be more favorable in terms of arming immune system against cancer, as observed in a recently published multi-cohort trial in patients with melanoma [71].

(4) As reflected by ongoing studies and summarized in Table 1, one of the strategies to increase clinical benefit is to combine checkpoint inhibitors with other systemic therapy. If it is already known that platinum-derived compounds are able to increase the release of TAAs and stimulate the immune response, this does not improve survival, probably due to a cytokine release syndrome. In order to overcome these mechanism of resistance, the addition of celecoxib to cisplatin and PD-L1 blockade has been proposed [54]. More recent studies demonstrated a potential role for the combination of immune checkpoint inhibitors and the DNA methyltransferase inhibitors, now commonly used in treatment of hematologic neoplasm [72]. In fact 5-azacytidine, in preclinical melanoma and NSCLC models, sensitizes cells to the check point inhibitors through an upregulation of immunoregulatory pathways [73,74]. In ovarian cancer, preclinical studies have shown that immune gene demethylation and expression after 5-azacytidine exposure is higher than other cell lines, such as breast and colorectal cancers [74]. Ovarian cancer is known to have an angiogenic phenotype [13]. From the preclinical evidence currently available, and currently under clinical evaluation, the association between checkpoint inhibitors and antiangiogenic drugs (Table 1) also appears reasonable. Indeed, it has been established that vascular endothelial growth factor (VEGF) has an immune suppressive effect on T cells activation and inversely correlates with TILs infiltration [13,15], Moreover, VEGR receptor 2 (VEGFR2) is selectively expressed in Treg CD4+FoxP3+ cells and in response to VEGF, immature dendritic cells acquire a pro-angiogenic phenotype and contribute to ovarian cancer progression [75,76,77,78].

(5) Interestingly, Chen and colleagues have shown that a soluble isoform of PD-L1 (sPD-L1) is detectable in sera from healthy humans with an enzyme-linked immunosorbent assay (ELISA) test. It is, therefore, likely that, in the presence of PD-L1 expressing cancer cells an increase of sPD-L1 may be possible. Considering that delayed diagnosis influence prognosis of ovarian cancer, it could be useful to assess a possible correlation between sPD-L1 levels and early detection of ovarian cancer [79].

In conclusion, ovarian cancer is an immunogenic disease and currently available data suggest a potential activity of immune checkpoint inhibitors. There are still some points to be addressed, such as the identification of reliable predictors of response, optimal clinical setting and finally, we need to understand whether these drugs give their best alone or in combination with other agents. To significantly move forward with immune therapy in EOC, it be will crucial to design smart clinical trials with appropriate endpoint selection and biomarker assessment.

## Figures and Tables

**Table 1:** Clinical trials with immune checkpoints inhibitors in ovarian cancer.

Type of Treatment	ID	Condition	Phase	Primary Outcome	Secondary Outcome	Status	Sponsor
Neoadjuvant Pembrolizumab + chemotherapy then Pembrolizumab maintenance therapy	NCT02520154	Advanced EOC/primary peritoneal/fallopian tube cancer	2	PFS		Not yet recruiting	M.D. Anderson Cancer Center
Pembrolizumab with first line platinum based chemotherapy followed by pembrolizumab manteinance therapy	NCT02766582	Suboptimally cytoreduced EOC/primary peritoneal/fallopian tube cancer	2	PFS	OS, QoL	Not yet recruiting	Medical College of Wisconsin
Niraparib + Pembrolizumab	NCT02657889	Advanced or metastatic triple-negative breast cancer or recurrent EOC	1–2	DLT, ORR	Safety, tolerability, DR, DCR, OS	Recruiting	Tesaro, Inc.
Chemotherapy + pembrolizumab and pembrolizumab as maintenance therapy	NCT02608684	Platinum-resistant recurrent EOC	2	ORR	PFS, time to progression, DR, OS	Recruiting	Cedars-Sinai Medical Center
Pembrolizumab	NCT02674061 (KEYNOTE-100)	Advanced recurrent EOC	2	ORR	DCR	Recruiting	Merck Sharp & Dohme Corp.
Dose Dense Paclitaxel with pembrolizumab	NCT02440425	Platinum-resistant recurrent EOC	2	PFS, safety	ORR, DCR, DR, OS	Recruiting	H. Lee Moffit Cancer Center and Research Institute
ACP-196 ± Pembrolizumab	NCT02537444 (KEYNOTE-191)	Platinum-sensitive recurrent EOC	2	ORR		Recruiting	Acerta Pharma BV
WT1 Vaccine and Nivolumab	NCT02737787	Recurrent EOCin CCR after chemotherapy	1	DLT		Recruiting	Memorial Sloan Kettering Cancer Center
Atezolizumab + Bevacizumab + Acetylsalicylic Acid	NCT02659384	Platinum-resistant recurrent ovarian cancer	2	PFS		Not yet recruiting	EORTC
Durvalumab + Paclitaxel and Carboplatin	NCT02726997	High-grade non-mucinousEOC, primary peritoneal or fallopian tube cancer	1–2	Pharmacokinetics	PFS, feasibility	Not yet recruiting	M.D. Anderson Cancer Center
Toll-like Receptor Agonist 8 Motolimod (VTX-2337) + Durvalumab	NCT02431559	Platinum-resistant recurrent EOC	1–2	MTD, PFS		Recruiting	Ludwig Institute for Cancer Research
Durvalumab + Olaparib or Cediranib	NCT02484404	Advanced solid tumors and recurrent EOC	1–2	Recommended dose, ORR		Recruiting	National Cancer Institute (NCI)
Durvalumab	NCT02764333	Platinum-resistant EOC	2	ORR		Recruiting	Memorial Sloan Kettering Cancer Center
Avelumab ± PLD Versus PLD Alone	NCT02580058	Platinum-resistant/refractory EOC	3	OS	ORR, DC, PFS, DR	Recruiting	Pfizer
avelumab in combination with and/or following platinum-based chemotherapy	NCT02718417	Previously untreated EOC	3	PFS	OS, maintenance PFS, ORR, DR	Not yet recruiting	Pfizer
Olaparib + Tremelimumab	NCT02571725	recurrent BRCA mutation-associated EOC	1–2	Recommended dose, ORR	PFS	Recruiting	New Mexico Cancer Care Alliance
Tremelimumab ± Olaparib	NCT02485990	recurrent or persistentEOC, fallopian tube or primary peritoneal carcinoma	1–2	Safety		Recruiting	Sidney Kimmel Comprehensive Cancer Center
Ipilimumab	NCT01611558	Recurrent platinum sensitive EOC	2	Safety	ORR	Active but not recruiting	Bristol-Myers Squibb
Nivolumab ± Ipilimumab	NCT02498600	Recurrent EOC/primary peritoneal/fallopian tube cancer	2	Objective tumor response	OS, PFS, safety	Suspended	National Cancer Institute (NCI)

EOC = epithelial ovarian cancer, OS = overall survival, PFS = progression free survival, DR = duration of response, DCR = disease control rate, QoL = quality of life, DLT = dose-limiting toxicities, ORR = overall response rate, CCR = complete clinical remission, MTD = maximum tolerated dose, PLD = pegylated liposomal doxorubicin.
